# Case Report: Pelvic mass and massive ascites as the first symptom in cervical adenocarcinoma: report of two cases and literature review

**DOI:** 10.3389/fonc.2023.1244202

**Published:** 2023-08-11

**Authors:** Mingwei Yuan, Yan Zhang, Kana Wang, Mingrong Xi

**Affiliations:** ^1^ Department of Obstetrics and Gynecology, West China Second University Hospital, Sichuan University, Chengdu, China; ^2^ Key Laboratory of Birth Defects and Related Diseases of Women and Children (Sichuan University), Ministry of Education, Chengdu, China; ^3^ Department of Pathology, West China Second University Hospital, Sichuan University, Chengdu, China

**Keywords:** case report, cervical adenocarcinoma, gastric-type endocervical adenocarcinoma (G-EAC), pelvic mass, ascites

## Abstract

Cervical adenocarcinoma accounts for 10%–25% of total cases of cervical carcinoma. But in recent years, the incidence of adenocarcinoma has risen both proportionally and absolutely. Clinically, most cervical adenocarcinoma show no symptom or present with abnormal uterine bleeding or vaginal discharge, similar to squamous cell carcinoma. What different about it is that cervical cytological testing demonstrates a high false-negative rate of cervical adenocarcinoma, potentially leading to the failure in detecting in early stage. This report presents two cases both with pelvic masses, and massive ascites served as the initial symptom, which is similar to the clinical symptom of ovarian cancer, but ultimately diagnosed with cervical adenocarcinoma through surgical specimens. There are few literature reports on this situation. Hence, a literature review also has been performed to improve the recognition for cervical adenocarcinoma presenting with pelvic masses and massive ascites, and to avoid misdiagnosis.

## 1 Introduction

Cervical carcinoma is the most common tumor in the female reproductive system in China. In the year 2020, approximately 604,127 cases of cervical carcinoma were detected, resulting in 341,831 deaths caused by this disease (1). Squamous cell carcinoma (SCC) represents the most prevalent type of cervical cancer, which is predominantly linked to human papilloma virus (HPV). With the introduction of national screening and the development of HPV vaccination, the occurrence of SCC has resulted in a decline during the past few decades (2). However, cervical adenocarcinoma represents 10%–25% of the total cervical carcinoma, both the proportional and absolute rates have exhibited an upward trend in recent decades (3–8). A study carried out in 13 European nations revealed at least 2% yearly rises in rates of cervical adenocarcinoma in Finland, the United Kingdom, Slovakia, and Slovenia. Positive trends, albeit not statistically significant, were observed in majority of the other countries (5).

In 2018, the International Endocervical Adenocarcinoma Criteria and Classification (IECC) established a classification system for endocervical adenocarcinomas, distinguishing them as either HPV-associated adenocarcinomas (HPVA) or non-HPV-associated adenocarcinomas (NHPVA) based on the presence or absence of features associated with HPV infection (9). The clinical symptom of these NHPVA patients are different from those of cervical squamous cell carcinoma, and cervical cytological testing exhibits a high false-negative rate, which may result in the misdiagnosis (5, 10, 11). Prior investigations have suggested an unfavorable prognosis for patients with NHPVA who present larger tumor sizes (12).

Pelvic masses and massive ascites commonly manifest as symptoms of malignant ovarian tumors and extremely rare occurrence in cervical cancer. Here, we reported two cases both with pelvic masses and massive ascites as the initial symptom, ultimately leading to the diagnosis of cervical adenocarcinoma through surgical specimens. In addition, we performed a PubMed search and review literature for the similar cases in the past 20 years, which are summarized in the current study (**Table 1**).

**Table 1:** Literature review of cervical carcinoma with pelvic mass and ascites as the first symptom from 2006.

Author (year)	Age	Symptom	Preoperative diagnosis	Clinical stage	Pathologic diagnosis	Surgical methods	Radiotherapy and chemotherapy	Postoperative follow-up
JamieL. McDowell (2021)	50	abdominal pain and distension, huge abdominal mass, obvious ascites	Ovarian or peritoneal malignancy	/	G-EAC	TAH, BSO, omentectomy, resection of the pelvic mass	systemic chemotherapy: cisplatin+paclitaxel+bevacizumab,external beam radiation therapy and vaginal brachytherapy	/
Seema Singhal (2007)	47	abdominal pain, right ovarian cyst, moderate ascites	right ovarian cyst	IV	cervical adenocarcinoma	first surgery: right salpingo-oophorectomy,second surgery: TAH, left salpingo-oophorectomy,omentectomy with staging	six courses chemotherapy: carboplatin+paclitaxel	18 months: no recurrence
Yi-Duen Huang (2006)	30	chest tightness, huge abdominal mass, massive ascites, pleural effusion	/	ovarian endometrioid adenocarcinoma stage IIIc, endocervical mucinous adenocarcinoma stage IA1	ovarian endometrioid adenocarcinoma, endocervical mucinous adenocarcinoma	TAH, BSO, BPLND, paraaortic lymph node sampling, bilateral infundibulopelvic ligament resection, appendectomy and omentectomy	six courses chemotherapy: Carboplatin+paclitaxel+Gemcitabine salvage chemotherapy: pegylated doxorubicin HClliposome	8 months: die

*TAH, total abdominal hysterectomy; BSO, bilateral salpingo-oophorectomy; BPLND, bilateral pelvic lymph node dissection.

## 2 Case presentation

### 2.1 Case 1

A 39-year-old Chinese Han woman went to a nearby hospital because of abdominal distension over 2 months. The initial local ultrasound and computed tomography (CT) scan showed “pelvic and abdominal masses and a large amount of hydrothorax and ascites.” After thoracic and abdominal drainage, no malignant tumor cells were found.

Then, the patient turned to our hospital for further evaluation. During the gynecological examination, a mass measuring approximately 10+ cm in diameter was located in the right pelvic cavity, while no abnormalities were detected in the vagina, cervix, and uterus. Additionally, there were evident ascites with a fluid wave. Cervical liquid-based cytological test was negative, and the hybrid capture 2 (HC2) was 235.05. The result of serum carbohydrate antigen-125 (CA-125) level was 146.9 U/ml, while other tumor markers were within normal ranges. Gynecological ultrasound (US) and enhanced pelvic computed tomography (CT) scan in our hospital suggested a pelvic mass approximately 10+cm suspected to originate from the right adnexa and free fluid in the pelvis and abdomen approximately 5.4cm and 10.1cm in depth, respectively. The chest computed tomography (CT) scan did not reveal any nodules. Since the onset of the disease, she had lost 5 kg in weight. Considering all the mentioned findings, the patient was admitted to our department with a diagnosis of pelvic mass suspected to ovarian malignancy.

During single-port exploratory laparoscopy, approximately 2,800 ml of pale yellow, mucinous ascites was observed; the pelvic mass was approximately 12 cm in diameter, completely occupying the entire pelvic cavity and originating from the right adnexa; and the uterus was enlarged with a smooth surface. On the surface of the rectum and its mesentery, there were scattered nodules approximately 0.2–0.5 cm in diameter. No obvious abnormalities were observed in the left fallopian tube, left ovary, omentum majus, appendix, mesentery, intestinal surface, left and right colon grooves, diaphragmatic surface, liver, or stomach. The laparoscopy score was 0 point. The intraoperative frozen-section examination of the right ovarian tissue showed an intermediate-grade Müllerian tumor. Due to the large size of the tumor, the exact nature of the tumor could not be determined. When the patient and her husband been informed of the intraoperative situation before surgery, they requested to perform a comprehensive staging surgery with laparotomy. During the laparotomy, the patient underwent resection of the large pelvic mass (including the appendix), total hysterectomy, left salpingo-oophorectomy, pelvic lymph node dissection, para-aortic lymph node sampling, and omentectomy. No visible tumor residues were left after surgery, achieving R0 resection. After the resection, a 1cm polyp was observed at the junction of cervix and uterine. Before discharge, the patient had a recurrence of hydrothorax and ascites.

**Figure 1:**
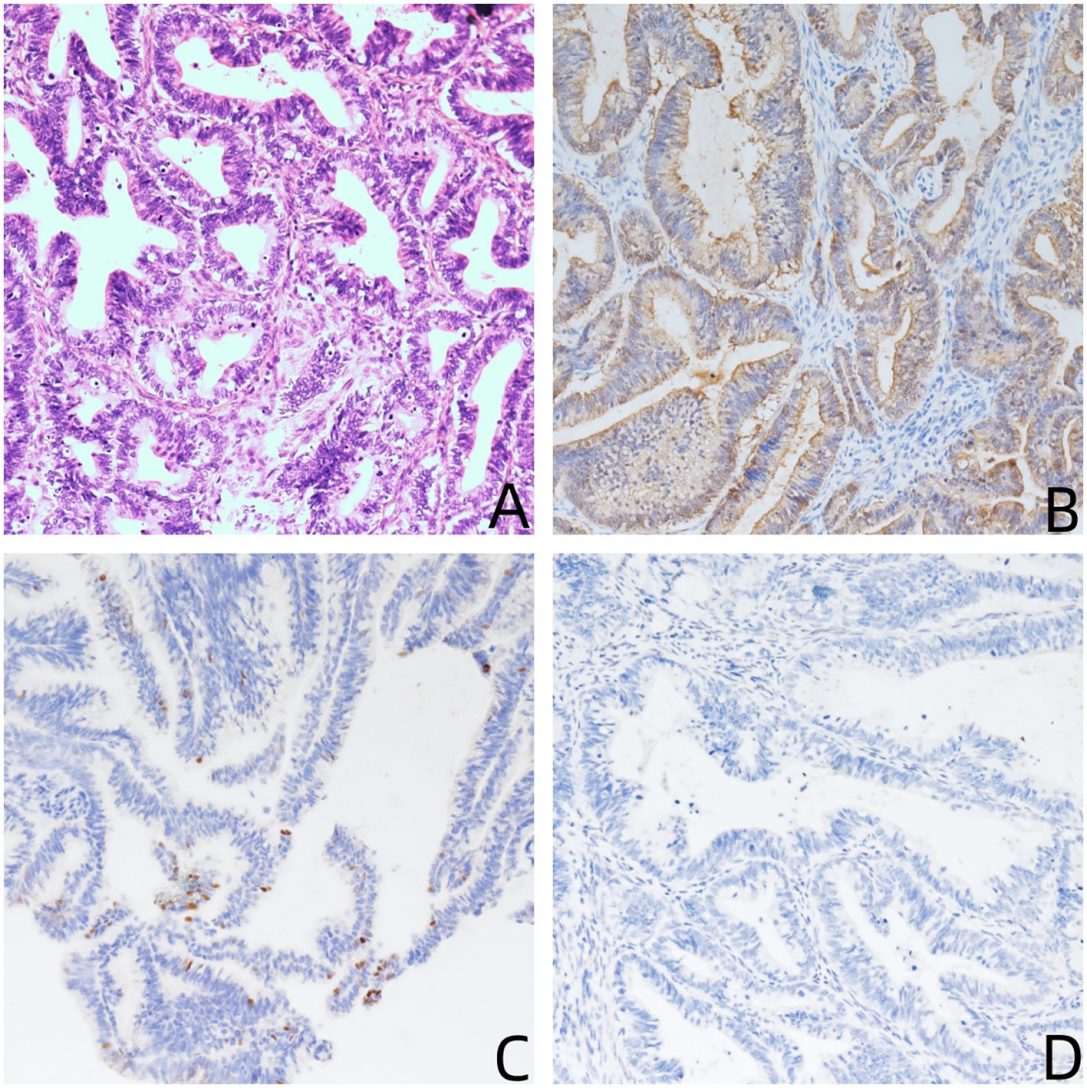
**(A)** Hematoxylin and eosin stain (200×): the tumor shows endocervical adenocarcinoma (common type); **(B–D)** immunohistochemical stains (200×) are positive for P16 **(B)**, P53 wild type **(C)**, and negative for WT-1 **(C)**.

The histological findings of the cervix revealed intermediate to high differentiation endocervical adenocarcinoma (common type/HPV-related type), penetrating superficial one-third layer of stroma; extending to the junction of the cervix and uterus; the lower segment of the uterus endometrium and shallow layer of the uterine wall; and spreading to the right ovary and omentum majus. No cancer metastases was discovered in the lymph nodes that were removed (0/42). The immunohistochemistry staining results of both the cervical and right ovarian metastatic cancers were P16(+++), P53 wild type (+), Muc-5(+++), CK7(+++), ER(−), PR(−), Muc-2(−),WT-1(−), and CK20(−), and the Ki67 proliferative index was 90% (**Figure 1**). High-risk HPV 16 test was positive.

Subsequently, the patient received six cycles of systemic chemotherapy including cisplatin, paclitaxel, and bevacizumab. Additionally, the patient received external beam radiation therapy and vaginal brachytherapy.

Follow-up for the patient involved regular clinical assessments, tumor marker tests, and CT scans of the chest and pelvis every 3 months. After a 12-month follow-up, the patient exhibited a positive response to the treatment, with no clinical or radiological evidence of recurrence.

### 2.2 Case 2

A 51-year-old Chinese Han woman went to a nearby hospital with complaints of abdominal distension persisting for over 10 days. The initial local ultrasound showed “a solid cystic mass measuring approximately 16 cm in pelvic along with massive ascites.” The patient had a medical history including psoriasis, surgeries for the removal of cervical polyps, and breast lobular hyperplasia.

Subsequently, the patient sought further evaluation in our hospital. During the gynecological examination, a soft, smooth, painless mass approximately 20 cm in diameter was located in the pelvic cavity, along with evident ascites, while no abnormalities were detected in the vagina, cervix, and uterus. Cervical liquid-based cytological test was negative. The results of tumor markers revealed the following results: CA-125, 34.9 U/ml; CA19-9, 10.2 U/ml; CEA, 0.6 ng/ml; and AFP, 2.7 U/ml. Gynecological ultrasound (US) showed a septated cystic mass with a size of 20 cm in pelvic and abdominal cavity and the cystic fluid was relatively clear, and some solid echoes were visible inside. A liquid dark area was found in the pelvic and abdominal cavity, with a maximum depth of approximately 8.0 cm. Multiple cystic masses were found in the cervix, with the largest diameter of 1.0 cm. Enhanced pelvic computerized tomography (CT) indicated a solid cystic mass, which suspected to be malignant (possibly from the left ovary), some nabothian cyst in the cervix, and a large amount of pelvic fluid. Gastroscopy showed chronic moderate superficial gastritis. Considering all the findings, the patient was admitted to our department with a diagnosis of a pelvic mass suspected to be ovarian malignancy.

During single-port exploratory laparoscopy, approximately 3,960 ml of pale yellow, mucinous ascites was observed, and the pelvic mass was approximately 20 cm in diameter originated from the left adnexa, composed of 9–10 solid cystic structures fused together with a thin wall and a smooth surface. There were some ruptures ranging from 0.5 to 2 cm. No obvious abnormalities were observed in the right fallopian tube, right ovary, pelvic lymph nodes, omentum majus, appendix, mesentery, intestinal surface, left and right colon grooves, diaphragmatic surface, liver, or stomach. The intraoperative frozen-section examination of the left adnexa tissue indicated a mucinous tumor with a wide range of borderline changes, accompanied by intraepithelial carcinoma. Due to the large size of the tumor, the exact nature of the tumor could not be determined. The patient and her husband had been informed of the intraoperative situation before surgery. They requested to perform a comprehensive staging surgery with laparotomy. During the laparotomy, the patient underwent resection of total hysterectomy, bilateral salpingectomy, pelvic lymph node dissection, para-aortic lymph node sampling, appendectomy, and omentectomy. No visible tumor residues were left after surgery, achieving R0 resection. After the resection, multiple cysts containing transparent mucus can be seen near the inner mouth of cervical canal.

**Figure 2:**
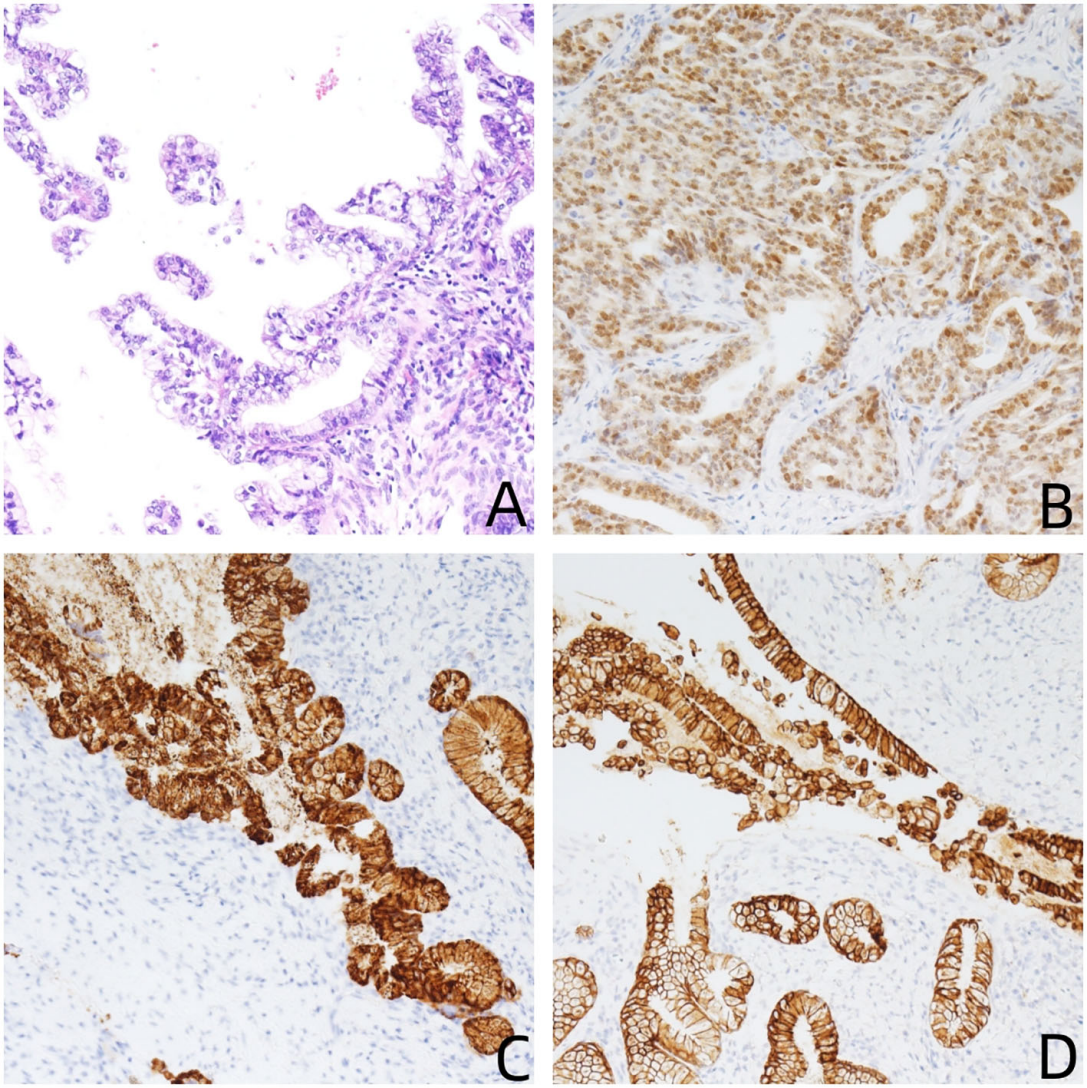
**(A)** Hematoxylin and eosin stain (200×): the tumor shows G-EAC; **(B–D)** immunohistochemical stains (200×) are focally positive for P53 muted type **(B)**, Muc-6 **(C)**, and CK7 **(D)**.

The histological findings of cervix revealed a high-grade differentiation mucinous adenocarcinoma of the cervix, specifically of the gastric type, penetrating more than one-half of the stroma, extending to the junction of cervix and uterus, uterus endometrium, and bilateral adnexa. Furthermore, adenocarcinoma cells were discovered in ascites. No cancer metastases were discovered in the lymph nodes that were removed (0/22). The immunohistochemistry (IHC) staining results of the cervix were CK7(+), P53 muted type(+), Villin(+), Muc-6(+), P16(+, focally), Pax-8(+, focally), Muc-5(+), ER(−), PR(−), CK20(−), and CDX-2, and the Ki67 proliferative index was 10% (**Figure 2**). In the left adnexa, metastatic mucinous adenocarcinoma was observed, and IHC results were CK7(+), P53 muted type(+), Villin(+), Muc-6(+), CK20(−), Pax-8(−), and the Ki67 proliferative index was 40%.

Subsequently, the patient received six cycles of systemic chemotherapy including cisplatin, paclitaxel, and bevacizumab. Additionally, the patient received external beam radiation therapy and vaginal brachytherapy.

Follow-up for the patient involved regular clinical assessments, tumor marker tests, and CT scans of the chest and pelvis every 3 months. After a 12-month follow-up, the patient exhibited a positive response to the treatment, with no clinical or radiological evidence of recurrence.

## 3 Discussion

Currently, liquid-based cytology examination is one preferred method for cervical cancer screening. Based on the results of liquid-based cytology, colposcopy-guided multiple-point biopsies are performed. If the biopsy results are inconclusive, cervical conization pathology is conducted. Cervical adenocarcinoma exhibits less pronounced nuclear atypia in exfoliated cells compared to squamous cell carcinoma. It originates from the deep cervical canal’s glandular epithelial cells and infiltrates the cervical stroma (13). In elderly women, cervical atrophy leads to the migration of the transformation zone and concealed lesions, often resulting in inadequate sampling. To minimize missed diagnoses, multiple-point cervical biopsies and cervical scraping are recommended, and if necessary, cervical conization or biopsies under hysteroscopy are performed. Liquid-based cytology has a higher rate of missed diagnoses for cervical adenocarcinoma, and both cases in this study did not show any significant abnormalities in cervical liquid-based cytology. In cases where pelvic masses coexist with cervical lesions, careful observation of the cervix is essential, with consideration given to cervical conization or hysteroscopic biopsies to avoid missed diagnoses.

Risk factors associated with endocervical adenocarcinoma (ECA) resemble those of squamous cell carcinoma (SCC), including multiple sexual partners, early age at first intercourse, prolonged use of oral contraceptives exceeding 10 years, hormonal replacement therapy, and obesity (14–16). In early stage or when the tumor is confined to the endocervical canal, clinical examination may not reveal noticeable abnormalities. ECA can manifest as an exophytic mass with a polypoid, papillary, or nodular appearance, or as an ulcerated lesion. In rare instances, it may lead to a “barrel-shaped” cervix, characterized by thickening of the cervical wall. Both cases were admitted due to the discovery of pelvic masses and thoracoabdominal fluid accumulation, without presenting typical clinical symptoms of cervical cancer, and they were diagnosed with ovarian metastasis from cervical cancer after surgery. Computed tomography (CT), magnetic resonance imaging (MRI), and occasionally positron emission tomography (PET)/CT should be utilized for accurate staging, particularly in patients with apparent locally advanced disease.

ECA is believed to originate from pluripotential subcolumnar reserve cells. Most cases of ECA develop within the transformation zone, with a smaller proportion located in the endocervical canal near the lower uterine. ECAs consist of a diverse group of tumors, often displaying a combination of different cell types and patterns. The assessment method of the 2018 International Endocervical Adenocarcinoma Criteria and Classification (IECC) involves the examination of nuclear atypia and apoptotic bodies under 200× magnification. This classification system helps distinguish between these two types of endocervical adenocarcinoma based on histopathological characteristics. If these features are observed, it indicates HPV-associated adenocarcinoma (HPVA). In cases where nuclear atypia and apoptotic bodies are not easily observed at low magnification, the adenocarcinoma is classified as non-HPV-associated (NHPVA). If focal HPVA features are observed under 200× magnification, the tumor is considered to have limited “HPVA features” and is provisionally categorized as NHPVA. Subsequently, the histological subtype is further differentiated based on the cytomorphological characteristics of the tumor cells (9). After reviewing the histopathological slides by pathologists, the cellular morphology and arrangement of the ovarian tumors in these two cases were found to be identical to those of the primary cervical lesions.

Current guidelines for cervical cancer recommend similar treatment approaches for both squamous cell carcinoma and adenocarcinoma. Early-stage cervical cancer is typically treated with either surgery or radiotherapy (17). However, it is important to note that there are differences in the biological behavior, treatment outcomes, and prognostic factors between cervical adenocarcinoma and squamous cell carcinoma (18).

Compared to cervical squamous cell carcinoma, cervical adenocarcinoma has higher rates of ovarian metastasis. In stage IB, the ovarian metastasis rate for adenocarcinoma is 3.72%, while for squamous cell carcinoma, it is 0.22%. In stage IIA, the rates are 5.26% for adenocarcinoma and 0.75% for squamous cell carcinoma. In stage IIB, the rates increase to 9.85% for adenocarcinoma and 2.17% for squamous cell carcinoma (19). A meta-analysis of five studies on surgically treated patients with early stage cervical cancer revealed a 5.27-fold higher incidence of ovarian metastases in adenocarcinoma compared to squamous cell carcinomas (20). In a retrospective review and autopsy findings of 42 women who died from cervical cancer, it was observed that patients with adenocarcinoma had a higher incidence of certain metastatic features compared to those with squamous cell carcinoma. Specifically, adenocarcinoma patients had a higher incidence of para-aortic node metastases (61.9% versus 30.0%), uterine corpus involvement (100% versus 60.0%), adrenal gland metastases (33.3% versus 0%), ascites (42.8% versus 9.5%), and hydrothorax (42.8% versus 14.3%). These differences were found to be statistically significant (p < 0.05) (17, 21).

However, these studies suggest that these factors may not differ significantly between the two types of cervical cancer at an early stage, such as in lymph node and ovarian metastases, and parametrial, uterine or vaginal extension, lymphovascular-space involvement (LVSI), and depth of invasion (22–24). The diagnostic criteria for ovarian metastasis of cervical adenocarcinoma, as proposed by Hovland et al. in 2010, include the resemblance of ovarian tumor cells in morphology and arrangement to the primary cervical lesion. The ovarian lesions predominantly involve the medulla, which contradicts the predominant involvement of the cortex seen in primary ovarian cancer. Additionally, the presence of lymph node involvement or infiltration in other sites does not align with the diagnostic criteria for Warren’s repetitive carcinoma (25).

Approximately 80%–90% of cervical adenocarcinoma cases are associated with high-risk HPV infection. However, among the minority of cervical adenocarcinomas not related to HPV, gastric-type endocervical adenocarcinoma (G-EAC) is the most common, comprising approximately 1%–3% of all cases (26). G-EAC was indeed first reported by a Japanese pathologist in 2007 (27). It is recognized as an extremely invasive malignancy with a lower 5-year disease-specific survival rate of only 30%, in contrast to the higher survival rate of 77% observed for HPVA (28). Diagnosis of G-EAC relies mainly on cervical cytology examination, cervical scraping, and conization. However, the diagnosis of early-stage G-EAC remains challenging, with a misdiagnosis rate as high as 34% (29) due to the lack of specific clinical features in G-EAC and the morphological similarities between early-stage G-EAC and benign lesions. Clinical symptom of G-EAC are nonspecific, primarily characterized by vaginal discharge and irregular vaginal bleeding, and G-EAC typically presents in advanced stages and tends to have pelvic spread, particularly involving the ovaries, peritoneum, omentum majus, and distant metastases (30). Morphologically, early stage G-EAC is characterized by uneven size and increased number of mucinous glands, branching patterns, mild dysplasia, infiltrative growth, and overlapping morphological features with HPV-related cervical adenocarcinoma (31).

Reliance solely on morphology for diagnosing cervical adenocarcinoma is difficult; therefore, the use of immunohistochemical (IHC) is crucial for an accurate diagnosis during the clinical pathological evaluation. In HPVA, the IHC for P16 is typically diffusely positive, while in G-EAC, P16 is generally negative or only focally positive. This difference in P16 staining pattern can be used as a helpful diagnostic marker to distinguish between these two subtypes of cervical adenocarcinoma. However, approximately 8.5% of patients may exhibit diffuse strong positivity for P16, resembling HPVA (31–35). Therefore, it requires a comprehensive analysis combining pathological morphology and HPV testing to distinguish them. Approximately 50% of G-EAC patients exhibit TP53 mutation expression (34), focally positive or diffusely positive for CK7, CDX2, CK20, and PAX8 and negative expression for ER, PR, and PAX2. PAX8 has a positivity rate of 68%–80% and can be helpful in distinguishing tumors originating from the pancreaticobiliary system (32). The Ki67 proliferative index in G-EAC is typically low, usually <40%. Additionally, it was observed that primary ovarian cancer commonly exhibits positive ER and PR staining, whereas specimens of ovarian metastasis from cervical cancer tend to show negative staining. In our study, both Case 1 and Case 2 displayed negative results of ER and PR, further confirming the diagnosis of cervical adenocarcinoma metastasis to the ovary.

## 4 Conclusion

Cervical adenocarcinoma has a rate of misdiagnosis. Both of these cases presented with pelvic mass and massive ascites, as the first symptoms was misdiagnosed with ovarian cancer, emphasizing the need for increased awareness regarding this condition. In patients with pelvic masses, ascites, and cervical lesions, deep cervical biopsy should be performed to exclude cervical adenocarcinoma. Emphasis should be placed on the three-stage management of cervical lesions, including cytology, colposcopy, and histological examination, especially in high-risk populations. Follow-up and regular reevaluation of patients with cervical lesions should be prioritized. Prompt intervention is necessary upon detection of any abnormalities. Adherence to standardized, individualized, and patient-centered principles in the diagnosis and treatment of cervical adenocarcinoma has the potential to greatly enhance prognosis.

## Data availability statement

The original contributions presented in the study are included in the article/supplementary material. Further inquiries can be directed to the corresponding authors.

## Ethics statement

Written informed consent has been obtained from the individual(s) for the publication of any potentially identifiable images or data included in this article.

## Author contributions

MY worked on the case and wrote the manuscript. YZ participated in the collection of pathological images. MX and KW had given many constructive suggestions for this paper. All authors contributed to the article and approved the submitted version.
